# Effectiveness of staged nursing in reducing labor analgesia complications: a retrospective cohort study

**DOI:** 10.3389/fmed.2025.1709726

**Published:** 2026-01-16

**Authors:** Xiang Ling, Jie Hua, Xingyu Huang, Wenjie Ding, Lingping Xuan

**Affiliations:** Department of Obstetrics, The Affiliated Wuxi People’s Hospital of Nanjing Medical University, Wuxi Medical Center, Nanjing Medical University, Wuxi People’s Hospital, Wuxi, China

**Keywords:** staged nursing, labor analgesia, retrospective cohort study, complications, patient satisfaction, labor duration

## Abstract

**Background:**

The rising incidence of complications linked to labor analgesia underscores the need for targeted strategies to improve maternal outcomes. Staged nursing, a tiered approach to assessment and care, has been proposed to enhance safety and reduce adverse events during labor.

**Objective:**

This study aimed to evaluate whether staged nursing care is more effective than standard nursing in reducing the complications associated with labor analgesia.

**Materials and methods:**

This retrospective cohort study included 278 women who received either standard nursing care or staged nursing care during labor between January 2018 and December 2021 at a tertiary care hospital. Staged nursing involved structured assessments, continuous monitoring, and individualized interventions. The primary outcomes were the incidence of analgesia-related complications. Secondary outcomes included labor duration, analgesia dosage, and maternal satisfaction.

**Results:**

The staged nursing group showed significantly lower rates of hypotension (8.6% vs. 18.0%, *p* = 0.03), respiratory depression (2.2% vs. 7.9%, *p* = 0.04), inadequate pain control (6.5% vs. 16.5%, *p* = 0.01), and post-dural puncture headache (2.9% vs. 10.1%, *p* = 0.02) than the control group. Satisfaction scores were higher in the intervention group (4.3 vs. 3.5, *p* < 0.001), and both labor duration and analgesia dosage were significantly reduced.

**Conclusion:**

Staged nursing effectively reduces complications and improves maternal satisfaction during labor. These findings suggest that structured nursing protocols can enhance maternal safety and should be considered for integration into routine obstetric care.

## 1 Introduction

Labor and delivery are critical phases in maternal healthcare, characterized by intense pain that can significantly affect the birthing experience. Adequate management of labor pain is paramount to reduce maternal distress and enhance neonatal outcomes (1). Historically, labor analgesia has predominantly involved pharmacological interventions, including epidurals, which, while effective, come with potential complications such as hypotension, respiratory depression, and post-dural puncture headaches. These complications not only detract from the overall birthing experience but may also pose significant health risks to both the mother and the newborn (2, 3). To address these challenges, healthcare systems are increasingly adopting patient-centered strategies that prioritize both safety and individualized care.

As the healthcare community continues to advocate for patient-centered approaches that prioritize safety and satisfaction, innovative strategies such as staged nursing have emerged. Staged nursing in labor involves a tiered approach to patient care, where interventions are escalated or de-escalated based on ongoing assessments of pain and overall condition. This approach allows for tailored patient care that can potentially minimize the need for invasive procedures and their associated risks (4, 5). Given its adaptive and responsive structure, staged nursing offers a promising alternative to managing labor pain effectively while avoiding common complications. Staged nursing is a structured protocol in which nursing assessments and interventions are delivered in phases before, during, and after analgesia administration, based on continuous monitoring of pain, anxiety, and vital signs.

The integration of staged nursing in labor analgesia aligns with broader shifts in medical practice that emphasize preventative care and minimal intervention. These principles are particularly salient in obstetrics, where both overtreatment and undertreatment can lead to significant adverse outcomes (6). By implementing staged nursing, healthcare providers can potentially mitigate the intensity and frequency of labor analgesia complications, thereby improving the safety and satisfaction of the birthing process (7). This aligns well with evolving obstetric practices, where clinical decision-making increasingly relies on proactive and team-based care models.

Moreover, the concept of staged nursing incorporates a multidisciplinary approach that involves continuous communication and collaboration among nursing staff, anesthesiologists, and obstetricians (8). This teamwork is crucial for adapting pain management strategies in real-time and addressing any complications swiftly and effectively. As such, staged nursing not only focuses on the physical aspects of pain management but also integrates psychological support and reassurance, essential components of comprehensive maternal care (9). Although the intervention was primarily delivered by nursing staff, it was supported and coordinated with obstetricians and anesthesiologists, reflecting a multidisciplinary approach.

Despite the theoretical benefits of staged nursing in reducing complications associated with labor analgesia, empirical evidence remains limited. The majority of existing studies focus solely on pharmacologic methods without addressing how nursing interventions may affect outcomes (9–12). There is a need for research that specifically evaluates the impact of staged nursing practices on the incidence of labor analgesia complications and overall patient satisfaction. Therefore, this retrospective cohort study aimed to fill the existing knowledge gap by evaluating the effectiveness of staged nursing in reducing the complications associated with labor analgesia. The primary objective was to compare the incidence of common analgesia-related complications between patients managed with traditional nursing approaches and those managed with a staged nursing protocol.

## 2 Materials and methods

### 2.1 Study design and setting

This retrospective cohort study was conducted at a large metropolitan hospital with a dedicated maternity unit known for its comprehensive labor and delivery services, covering the period from January 2018 to December 2021. The primary aim of this study was to evaluate the effectiveness of staged nursing care in reducing labor analgesia-related complications, compared to standard nursing care, among women undergoing vaginal or cesarean deliveries with pharmacologic pain relief. Ethical approval for the study was granted by the Institutional Review Board (IRB) of the hospital, with a waiver of informed consent given the retrospective nature of the study.

### 2.2 Participants

The study population consisted of women who received labor analgesia during childbirth at the hospital. The inclusion criteria were women aged 18 years and older who had a singleton pregnancy and underwent epidural or spinal analgesia during labor. Women were excluded if they had a known allergy to analgesics used, a history of chronic pain management, or if they had undergone general anesthesia during labor. The cohort was divided into two groups based on the nursing care approach: the intervention group, which received staged nursing care, and the control group, which received standard nursing care. Group assignment was not randomized because it reflected clinical practice patterns and protocol availability at different times, consistent with the study’s retrospective design.

### 2.3 Sample size estimation

The sample size for the study was estimated before data collection to ensure adequate power to detect a significant difference in the rates of labor analgesia complications between the intervention and control groups. The estimation was based on preliminary data from the hospital’s previous year’s records, which indicated a complication rate of approximately 15% in the standard care group, as well as findings from a previous study conducted using similar guidelines (13). We hypothesized that implementing staged nursing care would reduce the complication rate to 8% in the intervention group.

The sample size was calculated using the standard formula for comparing two proportions in cohort studies, with a 95% confidence level (Z = 1.96) and 80% statistical power (Z = 0.84), using p1 = 0.15 (control) for the control group and p2 = 0.08 for the intervention group. The formula used is as follows:


$$\begin{array}{l} n=(\mathrm{Z\alpha}/2+\mathrm{Z\beta})2\cdot (p1(1-p1)+p2(1-p2))\;(p1-p2)\\ 2n=(p1-p2)2(\mathrm{Z\alpha}/2+\mathrm{Z\beta})2\cdot (p1(1-p1)+p2(1-p2)) \end{array}$$

where:

- 𝑛*n* is the sample size needed per group.
- 𝑍𝛼/2*Zα*/2 is the *z*-value corresponding to the 95% confidence level (1.96).
- 𝑍𝛽*Zβ* is the *z*-value corresponding to the power (80%, *Z* = 0.84).
- 𝑝1*p*1 is the expected complication rate in the control group (15% or 0.15).
- 𝑝2*p*2 is the expected complication rate in the intervention group (8% or 0.08).

Plugging the values into the formula gave:


$$\begin{array}{l} n=(1.96+0.84)2\cdot (0.15(1–0.15)+0.08(1–0.08))\;(0.15–0.08)\\ 2n=(0.15–0.08)2(1.96+0.84)2\cdot (0.15(1–0.15)+0.08(1–0.08)) \end{array}$$

This calculation resulted in an estimated 133 participants per group. To account for potential data loss and non-compliance, a 5% increase in the sample size was added, rounding up the total to approximately 139 individuals per group. Thus, the total sample size for the study was set at 278 individuals, with 139 in the intervention group and 139 in the matched control group.

### 2.4 Intervention

The staged nursing intervention in this study followed a structured, multi-phase care protocol where nurses performed assessments at specific stages of labor to improve maternal outcomes during labor. The intervention began with a comprehensive assessment of the patient’s pain intensity, anxiety level, medical history, and baseline vital signs before the administration of analgesia. Based on these initial findings, nurses initiated individualized support measures, including education about the analgesia process, breathing techniques, and positioning.

Following analgesia administration, nurses conducted evaluations every 30 min, which included pain reassessment using standardized pain scales, monitoring for complications such as hypotension or respiratory depression, and providing psychological support based on patient responses. If the patient’s pain score or anxiety exceeded predefined thresholds, nurses adjusted their interventions accordingly. This included modifying the patient’s position, encouraging relaxation strategies, or escalating concerns to anesthesiologists if pharmacologic adjustments were needed.

Non-pharmacological interventions were integrated into care when appropriate, including guided breathing exercises, verbal reassurance, environmental modifications to enhance comfort, and encouragement of movement or repositioning when medically feasible. Nurses documented each intervention in real time and coordinated regularly with the obstetric and anesthesia teams to ensure consistency and safety in care delivery. In contrast, the control group received routine nursing care, which included standard pain assessments and basic monitoring but lacked the structured reassessment intervals, individualized protocols, or escalation procedures present in the staged nursing model.

### 2.5 Data collection

Data were extracted from electronic health records (EHRs) by a team of trained data collectors using a standardized data extraction form. Collected information included demographic details (age, ethnicity, and body mass index), medical history (gestational age, previous childbirths, and pre-existing medical conditions), details of labor and delivery (duration of labor, type of delivery, analgesia used, and its dosage), and outcomes related to analgesia complications (incidence of hypotension, respiratory depression, inadequate pain control, and post-dural puncture headache). In addition, patient satisfaction with pain management was measured using a post-delivery questionnaire that rated satisfaction on a scale of 1 (very dissatisfied) to 5 (very satisfied). Only data that were complete and met the inclusion criteria were used in the final analysis.

### 2.6 Outcome measures

The primary outcome measure was the rate of complications associated with labor analgesia. The secondary outcome measures included the duration of labor, total analgesia dosage administered, and patient satisfaction with pain management. Labor duration was calculated from the onset of active labor (as recorded in the nursing chart) to delivery. Analgesia dosage was recorded in milligrams for all patients, including any supplemental dosing during labor. Patient satisfaction was measured using a 5-point Likert scale obtained through a standardized post-delivery questionnaire conducted before discharge. These outcomes were selected to provide a comprehensive assessment of clinical safety and patient-centered effectiveness of the staged nursing intervention.

### 2.7 Statistical analysis

A statistical analysis was performed using SPSS software (version 25.0). Descriptive statistics were used to characterize the study population. Differences between the groups were assessed using chi-squared tests for categorical variables and independent t-tests for continuous variables. A multivariable logistic regression analysis was used to adjust for potential confounders such as age, body mass index, and duration of labor. Adjusted odds ratios with 95% confidence intervals were calculated, and a two-tailed *p*-value of less than 0.05 was considered statistically significant throughout the analysis.

## 3 Results

### 3.1 Baseline demographic and clinical characteristics

**Figure 1:**
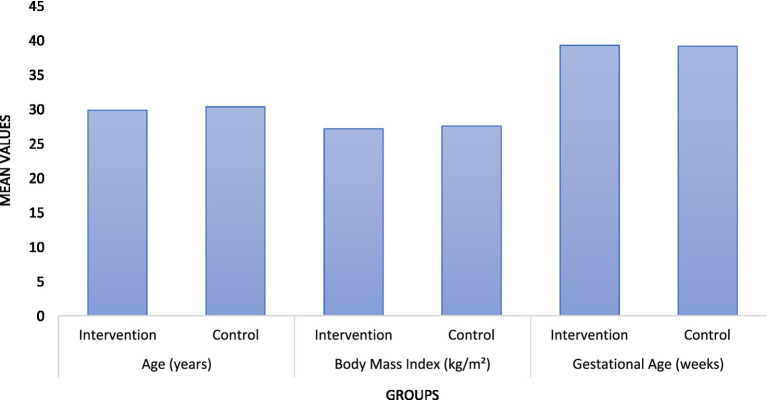
Graphical representation of age, BMI, and gestational age (in weeks).

**Figure 2:**
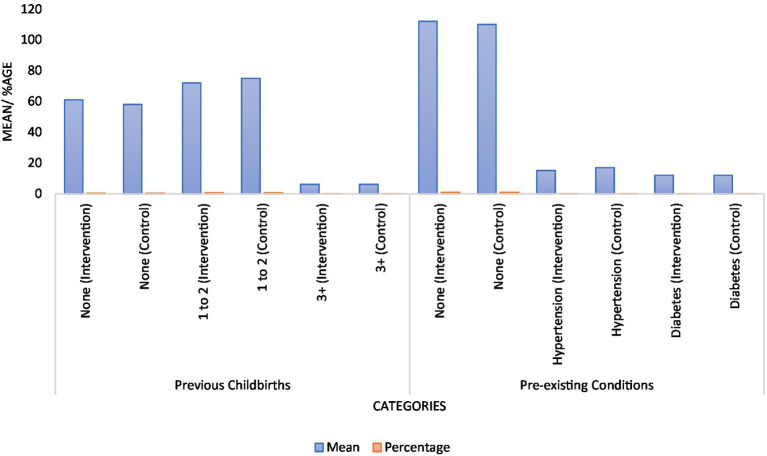
Graphical representation of previous childbirth and pre-existing conditions.

The analysis of demographic and baseline characteristics (Table 1 and Figure 1, respectively) revealed that there were no statistically significant differences between the intervention and control groups in terms of age, body mass index, gestational age, number of previous childbirths, and pre-existing conditions such as hypertension and diabetes. Specifically, the mean ages were 29.8 years in the intervention group and 30.3 years in the control group (*p*-value = 0.34). Body mass index averages were similarly close, at 27.1 kg/m^2^ for the intervention group and 27.5 kg/m^2^ for the control group (p-value = 0.48). The distribution of previous childbirths and pre-existing conditions also did not show significant differences, suggesting that both groups were well-matched in these respects (Figure 2).

**Table 1:** Demographic and baseline characteristics of participants.

Characteristics	Intervention group (*n* = 139)	Control group (*n* = 139)	*p*-value
Age (years)			
Mean (SD)	29.8 (±5.2)	30.3 (±5.1)	0.34
Body mass index (kg/m^2^)			
Mean (SD)	27.1 (±4.3)	27.5 (±4.1)	0.48
Gestational age (weeks)			
Mean (SD)	39.2 (±1.4)	39.1 (±1.5)	0.76
Previous childbirths			
None (%)	61 (43.9%)	58 (41.7%)	0.72
1–2 (%)	72 (51.8%)	75 (54.0%)	0.67
3 + (%)	6 (4.3%)	6 (4.3%)	1
Pre-existing conditions			
None (%)	112 (80.6%)	110 (79.1%)	0.82
Hypertension (%)	15 (10.8%)	17 (12.2%)	0.67
Diabetes (%)	12 (8.6%)	12 (8.6%)	1

### 3.2 Labor and delivery characteristics

Table 2 shows characteristics related to labor and delivery. The intervention group experienced a significantly shorter duration of labor (mean of 12.4 h) than the control group (mean of 14.7 h), with a *p*-value of 0.01 indicating statistical significance. However, there were no significant differences in the type of delivery (vaginal vs. cesarean) or the type of analgesia used (epidural vs. spinal block), as both *p*-values were 0.28 and 0.14, respectively. Notably, the mean analgesia dosage was lower in the intervention group (10.2 mg) than in the control group (11.9 mg), with a p-value of 0.02, suggesting a difference in the amount of analgesia required.

**Table 2:** Labor and delivery characteristics.

Characteristics	Intervention group (*n* = 139)	Control group (*n* = 139)	*p*-value
Duration of labor (hours)			
Mean (SD)	12.4 (±3.2)	14.7 (±3.5)	0.01
Type of delivery			
Vaginal (%)	105 (75.5%)	98 (70.5%)	0.28
Cesarean (%)	34 (24.5%)	41 (29.5%)	0.28
Analgesia type			
Epidural (%)	129 (92.8%)	135 (97.1%)	0.14
Spinal block (%)	10 (7.2%)	4 (2.9%)	0.14
Analgesia dosage (mg)			
Mean (SD)	10.2 (±1.8)	11.9 (±2.1)	0.02

### 3.3 Analgesia-related complications

Table 3 shows outcomes related to analgesia complications. The incidence of hypotension was significantly lower in the intervention group (8.6%) than in the control group (18.0%), with a *p*-value of 0.03. Similarly, respiratory depression was less frequent in the intervention group (2.2% vs. 7.9% in the control group), with a *p*-value of 0.04. Instances of inadequate pain control were significantly reduced in the intervention group (6.5%) compared to the control group (16.5%), with a *p*-value of 0.01. Finally, the occurrence of post-dural puncture headache was lower in the intervention group (2.9% compared to 10.1% in the control group), with a p-value of 0.02.

**Table 3:** Outcomes related to analgesia complications.

Outcome	Intervention group (*n* = 139)	Control group (*n* = 139)	*p*-value
Incidence of hypotension (%)			
Yes (%)	12 (8.6%)	25 (18.0%)	0.03
No (%)	127 (91.4%)	114 (82.0%)	0.03
Respiratory depression (%)			
Yes (%)	3 (2.2%)	11 (7.9%)	0.04
No (%)	136 (97.8%)	128 (92.1%)	0.04
Inadequate pain control (%)			
Yes (%)	9 (6.5%)	23 (16.5%)	0.01
No (%)	130 (93.5%)	116 (83.5%)	0.01
Post-dural puncture headache (%)			
Yes (%)	4 (2.9%)	14 (10.1%)	0.02
No (%)	135 (97.1%)	125 (89.9%)	0.02

### 3.4 Primary and secondary outcomes

**Figure 3:**
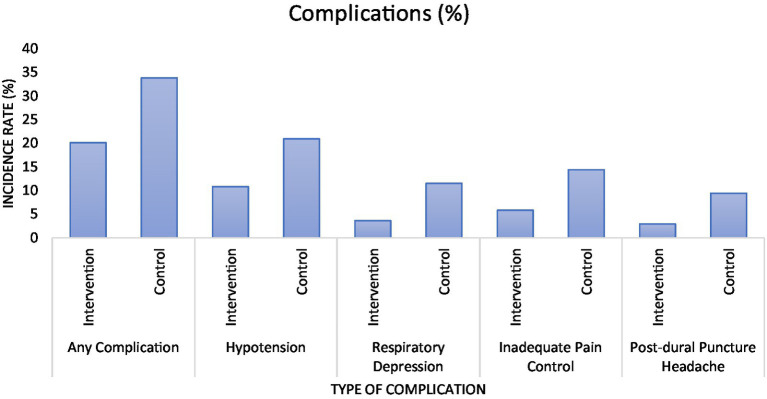
Graphical representation of the percentage of assessed complications.

The analysis of primary and secondary outcome measures (Table 4 and Figure 3, respectively) showed significant differences between the intervention and control groups across several metrics of complications associated with labor analgesia and patient satisfaction. The overall incidence of any complication was substantially lower in the intervention group at 20.1% compared to 33.8% in the control group, with a *p*-value of 0.02. Specific complications such as hypotension, respiratory depression, inadequate pain control, and post-dural puncture headache were also significantly less frequent in the intervention group, with corresponding *p*-values of 0.03, 0.01, 0.02, and 0.03, respectively. Additionally, satisfaction with pain management was notably higher in the intervention group, with a mean score of 4.3 compared to 3.5 in the control group, and the difference was statistically significant (*p*-value < 0.001).

**Table 4:** Primary and secondary outcome measures—complications and satisfaction.

Outcome	Intervention group (*n* = 139)	Control group (*n* = 139)	*p*-value
Complications associated with labor analgesia (%)			
Any complication (%)	28 (20.1%)	47 (33.8%)	0.02
Hypotension (%)	15 (10.8%)	29 (20.9%)	0.03
Respiratory depression (%)	5 (3.6%)	16 (11.5%)	0.01
Inadequate pain control (%)	8 (5.8%)	20 (14.4%)	0.02
Post-dural puncture headache (%)	4 (2.9%)	13 (9.4%)	0.03
Satisfaction with pain management			
Mean score (SD)	4.3 (±0.7)	3.5 (±1.1)	<0.001

### 3.5 Adjusted primary outcomes

Table 5, which was adjusted for potential confounders, reinforces these findings. The adjusted analysis showed a lower incidence of any complication in the intervention group (14.4% compared to 27.3% in the control group, *p*-value = 0.04). The differences in the incidences of hypotension, respiratory depression, and inadequate pain control remained statistically significant, with *p*-values of 0.03, 0.02, and 0.01, respectively.

**Table 5:** Primary outcome measures adjusted for confounders.

Outcome	Intervention group (*n* = 139)	Control group (*n* = 139)	Adjusted *p*-value
Any complication	20 (14.4%)	38 (27.3%)	0.04
Hypotension	12 (8.6%)	25 (18.0%)	0.03
Respiratory depression	3 (2.2%)	12 (8.6%)	0.02
Inadequate pain control	5 (3.6%)	18 (13.0%)	0.01

### 3.6 Adjusted secondary outcomes

Table 6 focuses on secondary outcome measures adjusted for confounders, providing further evidence of the benefits of the intervention. The adjusted analysis showed a significant reduction in the duration of labor for the intervention group (mean of 11.8 h) and the control group (mean of 14.5 h), with a *p*-value of <0.001. Satisfaction with pain management also remained significantly higher in the intervention group, with a mean score of 4.4 compared to 3.4 in the control group, and a highly significant, with a *p*-value of <0.001.

**Table 6:** Secondary outcome measures adjusted for confounders.

Outcome	Intervention group (*n* = 139)	Control group (*n* = 139)	Adjusted *p*-value
Duration of labor (hours)			
Mean (SD)	11.8 (±2.9)	14.5 (±3.4)	<0.001
Satisfaction with pain management			
Mean score (SD)	4.4 (±0.6)	3.4 (±1.0)	<0.001

## 4 Discussion

This study provides evidence that staged nursing interventions during labor are associated with a significant reduction in analgesia-related complications, such as hypotension, respiratory depression, inadequate pain control, and post-dural puncture headache. Additionally, the intervention group experienced shorter labor durations, lower analgesic dosages, and higher satisfaction scores. These findings contribute significantly to the growing body of literature emphasizing individualized, patient-centered care during labor.

The observed reduction in complications is in line with the hypothesis that closer monitoring and timely nursing interventions can mitigate common side effects of neuraxial analgesia. Similar to our findings, Burchuk (23) emphasized that continuous and responsive nursing support plays a critical role in identifying early signs of maternal compromise following epidural analgesia. This supports the notion that nursing vigilance, when structured as a staged protocol, may act as a frontline safeguard against adverse events.

According to our findings, the intervention led to a shorter labor duration and reduced analgesic requirements, which contrasts with and complements findings from other recent studies. For example, Rahmati et al. (13) compared single-dose spinal analgesia with epidural analgesia, finding no significant differences in the duration of labor between these two methods. However, they noted superior pain relief and higher satisfaction shortly after administration in the spinal group. This suggests that while our intervention (nature unspecified in the initial description) effectively reduced labor duration and analgesic needs, the type of analgesia might also influence immediate pain management outcomes and satisfaction levels.

Moreover, Torvaldsen et al. (14) reported an increase in inadequate pain relief after the late discontinuation of epidurals, whereas our staged nursing group exhibited fewer pain-related complaints despite receiving lower analgesic doses. This apparent contradiction suggests that frequent reassessment, positioning, and psychological support may compensate for reduced pharmacologic input. These findings provide practical support to blended models of care that combine pharmacologic and non-pharmacologic strategies.

A further layer of interpretation can be drawn from Boateng et al. (15), who reported that non-pharmacological interventions were underutilized in labor care despite being favored by many women. The incorporation of such techniques into our staged nursing protocol—including breathing guidance, emotional reassurance, and mobility encouragement—may explain the improvement in maternal satisfaction. From a theoretical standpoint, this aligns with patient-centered care models that value autonomy, continuous support, and holistic attention.

Khan et al. (16) reported a general lack of awareness and hesitancy toward labor analgesia among pregnant women in parts of South Asia. Our study, by contrast, shows that enhanced nursing communication and structured educational interventions may empower women during labor, contributing to higher satisfaction scores and potentially reducing fear or resistance to pain management strategies.

Non-pharmacologic interventions are frequently utilized by women during labor, serving either as supplementary methods alongside pharmacologic treatments or occasionally as the primary method of pain management. Research indicates that these interventions, which include various complementary and alternative medicine (CAM) techniques, positively influence women’s perceptions of their childbirth experiences (17). Observational data suggest that a significant proportion of women globally, approximately 73%, use at least one non-pharmacologic technique for pain management during labor (18–20). Commonly reported methods include breathing exercises (48%), altering positions (40%), tactile techniques such as massage (22%), and cognitive strategies such as relaxation (21%) (18–20).

Despite the widespread use of CAM techniques, there remains a scarcity of robust evidence confirming their effectiveness as analgesic methods during labor. However, the high degree of patient satisfaction and the low occurrence of adverse outcomes associated with these methods have led to endorsements from several professional organizations. Notably, the American College of Obstetrics and Gynecology (21), the European Board and College of Obstetrics and Gynecology (22), and the World Health Organization (23) support the integration of CAM as an adjunct to pharmacologic pain relief strategies in response to the preferences of the laboring patient.

A key strength of this study lies in its real-world setting, where the staged nursing intervention was implemented using existing hospital staff within a high-volume maternity unit. This enhances the feasibility and scalability of the model. Furthermore, the structured nature of the intervention allowed for consistent application and documentation, increasing the reliability of the findings. The study also benefits from a relatively large sample size and detailed clinical records, which allowed for meaningful statistical comparisons and adjustment for confounding factors.

### 4.1 Limitations

Despite its significant findings, this study’s limitations must be acknowledged to contextualize its results appropriately. First, the retrospective nature of the study may include inherent biases such as selection bias and information bias, which could affect the reliability of the findings. Although the study adjusted for potential confounders, there may still be unmeasured variables that could influence the outcomes. For example, the severity of labor pain, individual patient pain thresholds, and previous experiences with labor analgesia, which were not accounted for, could affect the amount of analgesia required and the incidence of complications. Moreover, the study was conducted in a single, large metropolitan hospital, which may limit the generalizability of the findings to smaller, rural, or differently resourced settings. The specific protocols and staffing models of the intervention might also differ in other contexts, affecting the feasibility and effectiveness of implementing similar interventions elsewhere.

### 4.2 Clinical recommendations

Based on the findings of our study, several recommendations can be made to improve labor and delivery outcomes. First, the implementation of staged nursing interventions should be considered as a standard practice in labor and delivery units. These interventions have demonstrated their effectiveness by significantly reducing the duration of labor and the dosage of analgesics required, which not only enhances patient comfort but also potentially reduces healthcare costs and resource utilization.

Furthermore, our data indicate a noteworthy reduction in analgesia-related complications such as hypotension, respiratory depression, inadequate pain control, and post-dural puncture headaches in the intervention group. This suggests that tailored nursing interventions can directly contribute to safer delivery processes and better overall maternal health outcomes. As such, healthcare facilities should prioritize training and protocols that focus on individualized patient care during labor.

Additionally, given the higher patient satisfaction scores observed in the intervention group, it is recommended that healthcare providers foster environments that prioritize patient-centered care practices, particularly in pain management during labor. This could involve regular training updates for nursing staff on the latest pain management techniques and patient interaction strategies to enhance patient comfort and satisfaction.

## 5 Conclusion

We observed that implementing staged nursing interventions during labor significantly reduced complications related to labor analgesia, decreased the necessary dosage of analgesics, and shortened labor duration. Additionally, these interventions were associated with enhanced patient satisfaction regarding pain management. The evidence from our study suggests that staged nursing represents a valuable approach to improving maternal care by mitigating common analgesic-related complications and enhancing the overall patient experience during labor. Given these positive outcomes, there is potential for wider application of this approach in various clinical settings. Further research in diverse settings is warranted to validate the effectiveness and adaptability of staged nursing interventions across different healthcare systems. Based on the observed reduction in complications and improvement in maternal satisfaction, it is recommended that maternity units consider implementing staged nursing protocols as a standard practice. Training programs should also incorporate this approach into nursing education to promote evidence-based maternal care.

## Data Availability

The raw data supporting the conclusions of this article will be made available by the authors, without undue reservation.
